# Neotendon infilling of a full thickness rotator cuff foot print tear following ultrasound guided liquid platelet rich plasma injection and percutaneous tenotomy: favourable outcome up to one year

**DOI:** 10.12688/f1000research.2-23.v1

**Published:** 2013-01-24

**Authors:** Arockia Doss

**Affiliations:** 1 Image Guided Therapy Clinic, Nedlands, 6009, Australia

## Abstract

This is a case report on excellent clinical outcome and neotendon infilling at one year follow up in a degenerative rotator cuff full thickness tear following percutaneous tenotomy and platelet rich plasma injection.

## Introduction

Rotator cuff tears are an increasingly common cause of morbidity in the aging population. There are surgical and non surgical management options including open or arthroscopic procedures, percutaneous corticosteroid injections, physiotherapy for rotator cuff tears. It is well accepted that there is a poor outcome in degenerative rotator cuff tears after surgery ^1^. This report is based on the clinical and imaging outcome in a patient who received platelet rich plasma (PRP) and percutaneous tenotomy treatment for a full thickness supraspinatus tear.

## Case report

A 73 year old right hand dominant active lady complained of bilateral shoulder pain for about two months and did not respond to two ultrasound guided subacromial subdeltoid corticosteroid injections. Her shoulder injury occurred whilst she had been caring for her husband and working on their farm. In the past she had recovered from non-Hodgkin’s lymphoma and currently was on antihypertensive medication (candesartan cilexetil once a day). She had led a physically active country life style prior to the presentation of the problem and had never smoked in her life.

**Figure 1.**
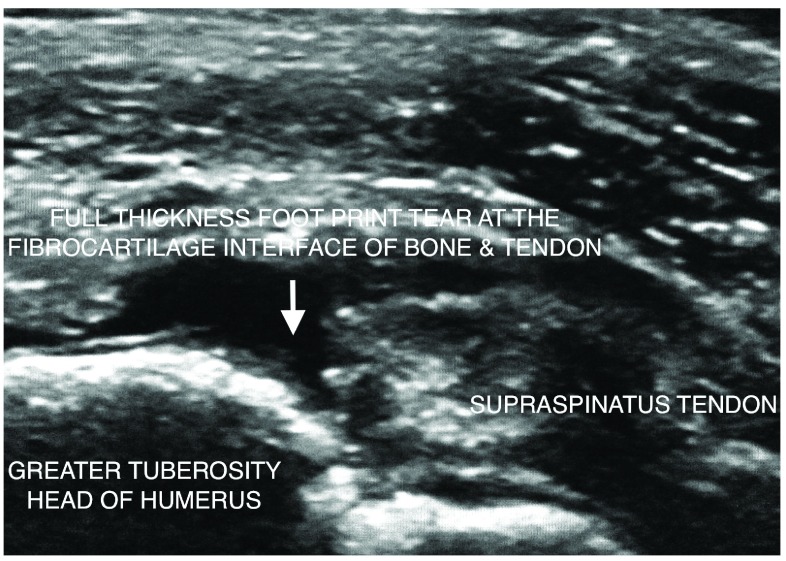
Long axis ultrasound image shows a full thickness footprint supraspinatus tear superimposed on degenerative tendinosis. Tear outline is well appreciated from distension of the peritendinous and subdeltoid bursal space during percutaneous treatment with tenotomy and liquid platelet rich plasma.

**Figure 2.**
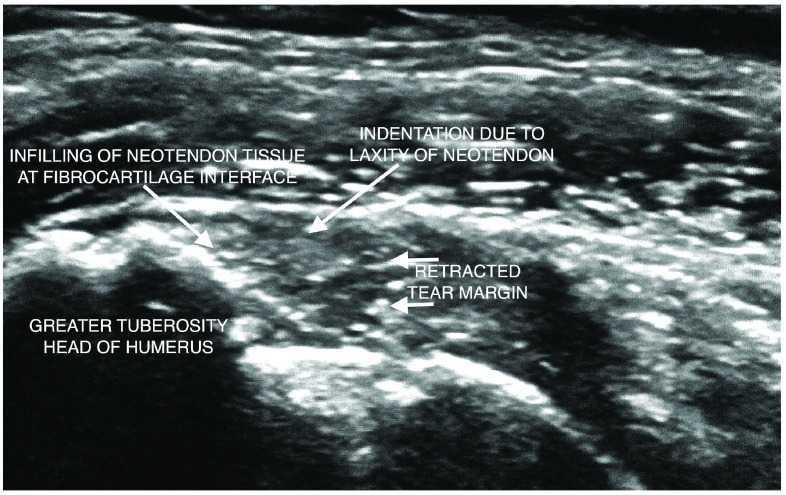
At follow up there is neotendon infilling in place of the tear defect. The lateral tear margin has retracted with mild interval attrition of the supraspinatus tendon. At the time of this follow up study the patient had a dramatic return to a completely pain free state and had full range of movement with no dysfunction.

At presentation there was painful limitation of right sided shoulder abduction to less than 90 degrees. Ultrasound documented a 9mm × 14mm partial width full thickness footprint tear of the anterior to mid right supraspinatus with tendinosis of most of the tendon and enthesopathy at the greater tuberosity of the humeral head ( Figure 1). A plain radiograph of the right shoulder showed a down-sloping Type 2 acromion. There was mild wasting of the right supraspinatus muscle. A diagnosis of a recent footprint tear superimposed on degenerative supraspinatus tendon with mild muscle atrophy was made. Following written informed consent from the patient, 8ml of autologous unclotted blood was venesected and centrifuged for 5 minutes at about 3000 rotations per minute in a special tube for PRP preparation (BCT, REGEN Labs, Switzerland). 4 to 5ml of liquid PRP was injected through a 22g 5cm long needle into the tear and its margins with simultaneous percutaneous tenotomy directed into the footprint of the anterior facet of the greater tuberosity under direct ultrasound imaging control (GE Logic 9, 9MHz probe). 5ml 1% lignocaine was injected into the superficial soft tissues, subacromial bursa and the supraspinatus tear for local anaesthetic purposes. The shoulder was placed in a sling with 90 degree elbow flexion for 7 days. Physiotherapy was commenced at 4 to 5 weeks post PRP with a home exercise program. At 8 weeks follow up post PRP the patient verbally reported a marked reduction in pain with improvement in shoulder movement. At the 7 and 10 month follow up there was complete relief from pain with full range of movement of the right shoulder and she was able to lift bags of potting mix in her farm. At the 10 month follow up, ultrasound (GE Logic 9, 9MHz probe) performed by the author showed a near complete echogenic infilling obliterating the tear defect. The lateral margin of the tear merged with this neotendon tissue with mild medial retraction ( Figure 2). She was completely pain free at a follow up 1 year after PRP injection.

## Discussion

PRP has gained increasing popularity in orthopaedic and musculoskeletal medicine in the last few years despite the lack of large volume data and high quality studies. Aside from its autologous nature, the rationale for the use of PRP is on the basis of its anti-inflammatory properties ^2^. Application of PRP in treating degenerative rotator cuff lesions is made on the basis of its role in the regulation of matrix gene expression and cell proliferation ^3^. PRP has also shown regenerative effects in an animal model of meniscal fibrocartilage tears ^4^ and this supports the use of PRP at the footprint of the supraspinatus insertion where there is fibrocartilagenous tissue at the bone-tendon interface.

This report documents imaging evidence of the formation of neotendon tissue in a patient who experienced complete resolution of symptoms from a full thickness partial width supraspinatus footprint tear. Symptom resolution at the time of manuscript preparation has lasted for 12 months post PRP injection. The precise histology of the neotendon infilling is of uncertain nature, although it is possible that this is fibrovascular tissue ^5^. Fibrovascular tissue scars at the bone-tendon interface are prone to failure and it remains to be seen if this patient’s relief persists in the long term. The favourable outcome cannot be entirely attributed to PRP. The possible influences of the percutaneous tenotomy, placebo effect, diet, and physiotherapy have to be taken into account. This report serves as anecdotal evidence that PRP does offer an alternative treatment option in some cases of full thickness rotator cuff tears.

Recent papers have contradicted the positive effect of PRP in rotator cuff tears. Two randomized controlled trials with 79 patients and another with 88 patients comparing PRP fibrin matrix (PRFM) versus control on rotator cuff tendon healing showed no demonstrable differences on tendon healing and clinical rating scales ^1, 6^. Bergeson *et al.* also showed similar results with PRFM in at risk rotator cuff tears ^7^. These studies used a semisolid implant material that had to be delivered through the arthroscope cannula. This implant was left at the bone tendon interface and may have resulted in a space occupying effect in addition to an unfavourable biological milieu with increased inflammatory mediators. PRP delivered as a semisolid plug in the form of PRFM through arthroscopic cannula is a different product dissimilar to liquid PRP preparations that are injectable under ultrasound guidance.

Routine or repeated corticosteroid injections into the subacromial bursa are controversial given that corticosteroid has a catabolic effect that may be harmful to already degenerative tendon tears. Repeated cortisone injections are questionable given that studies have documented an increased loss of bone mineral density after corticosteroid injections in postmenopausal women ^8, 9^ with the potential to increase the risk of fractures in this group of patients. In addition corticosteroid does not have a role in the healing cascade of degenerative tears ^10^. Although there is no doubt that in some instances corticosteroid injection will be needed due to individual circumstances, the widespread use of this approach deserves a rethink on the merits and disadvantages.

Clearly the routine use of PRP in rotator cuff tears is not recommended for all patients on the basis of this single case report. Until further high quality studies are available, use of PRP in rotator cuff tears may be reserved for recalcitrant pain in individuals with high compliance to post injection rehabilitation and in those where close clinical follow up and documentation of clinical outcome including any adverse events would be possible.

Further studies are urgently needed on the ultrasound guided percutaneous use of liquid PRP in degenerative rotator cuff tears. Questions remain on the role of PRP in rotator cuff tears. Is there a role for liquid PRP injection into cuff tears under imaging guidance prior to rotator cuff surgery? Does ultrasound guided PRP injection into the rotator cuff prior to surgery improve the outcome of surgical repair in comparison to subacromial subdeltoid bursal corticosteroid injection? Would PRP alone suffice in some patients and preclude the need for surgery thus reducing healthcare costs?

As far as the author is aware there in no similar published report. This case report documents that liquid PRP may play a favourable role in the stabilization of a full thickness supraspinatus insertional tear with an excellent clinical outcome and fuels the debate on the evolving role of autologous PRP in rotator cuff tears.

## Consent

Written informed consent for publication of clinical details and clinical images was obtained from the patient.
