# Supplementary material for: Neotendon infilling of a full thickness rotator cuff foot print tear following ultrasound guided liquid platelet rich plasma injection and percutaneous tenotomy: favourable outcome up to one year
Source: F1000Res. 2013 Jan 24;2:23. [Version 1] doi: 10.12688/f1000research.2-23.v1 (PMC4015560; doi:10.12688/f1000research.2-23.v1)
Supplement: Peer review history

# Peer review: Referee response for version 1

**Published:** 2014-03-21

**Authors:** Marco Patruno

**Affiliations:** 1 Department of Comparative Biomedicine and Food Science, University of Padova, Padua, Italy

In my opinion the author of this case report describes the results well, although I do agree with Elizaveta Kon that also including MRIs would have improved the quality of the paper. The real action of PRP is still under debate, and the scientific community asks for stringent methods and careful evaluations, even for a single case study. I suggest the author increases the number of patients and improves the quality of the results in future studies concerning PRP.

I have read this submission. I believe that I have an appropriate level of expertise to confirm that it is of an acceptable scientific standard.

# Peer review: Referee response for version 1

**Published:** 2014-01-27

**Authors:** Nicola Maffulli

**Affiliations:** 1 Institute for Science and Technology in Medicine, Keele University, Keele, UK

This article bears witness to how much we fall in love with novelties, and how much we, as a scientific community, do not know yet about a fashionable autologous blood product.

This case report is now one year old, and the situation in this field remains unchanged: randomised controlled trials show in a fairly unequivocable fashion that PRP use is at best dubious, and nevertheless case series report success.

This should make us think, and use strict stringent scientific methods to plan and evaluate new technologies.

I have read this submission. I believe that I have an appropriate level of expertise to confirm that it is of an acceptable scientific standard, however I have significant reservations, as outlined above.

# Peer review: Referee response for version 1

**Published:** 2013-02-06

**Authors:** Elizaveta Kon

**Affiliations:** 1 Biomechanics Laboratory, Rizzoli Orthopaedic Institute, Bologna, Italy

Literature concerning PRP use in rotator cuff pathology is mainly oriented towards intra-operative use of this biological strategy. However, recently, a double blinded randomized controlled trial on 39 patients has been published ( Rha DW *et al. *(2012) Comparison of the therapeutic effects of ultrasound-guided platelet-rich plasma injection and dry needling in rotator cuff disease: a randomized controlled trial). This article is a case report on the same topic, with all the scientific limitations related to the nature of such kind of article. At the present moment, also considering the controversies arisen on PRP application in tendon pathology, we need well designed high quality trials to assess the efficacy of this treatment option. 

The article is written in a fair manner without big methodological bias. However method is not only how you did what you did but also what you could have done better. Of course case reports provide poor evidence and it is impossible to rely just on findings from this kind of study. The author of the present study should have used some clinical scores (there are many available for the shoulder) to document outcome over time, MRI pre- and post-treatment should be added to better assess tendon healing and the features of PRP used should be discussed as this is one of the crucial points of current debate on PRP application. These changes could improve the scientific value of this case report, and it is important to be exhaustive when you have a single patient examined.

I have read this submission. I believe that I have an appropriate level of expertise to confirm that it is of an acceptable scientific standard.
